# Resistance of Scratched Fused Silica Surface to UV Laser Induced Damage

**DOI:** 10.1038/s41598-019-46048-4

**Published:** 2019-07-24

**Authors:** Hui Ye, Yaguo Li, Qiao Xu, Chen Jiang, Zhonghou Wang

**Affiliations:** 1 School of Mechanical Engineering, University of Shanghai for Science and Technology, Shanghai, 200093 China; 2 Research Center of Laser Fusion, China Academy of Engineering Physics, Mianyang, 621900 China

**Keywords:** Mechanical engineering, Applied optics

## Abstract

Scratches in fused silica are notorious laser damage precursors to UV laser damage initiation. Ductile and brittle scratches were intentionally generated using various polishing slurries. The distribution, profile and the dimension of scratches were characterized. The damage resistance of polished surfaces was evaluated using raster scanning damage testing protocol. The results show that both ductile and brittle scratches greatly increase area proportion of laser damage about one to two orders of magnitude relative to unscratched surface and brittle scratches are more deleterious. Moreover, finite difference time domain (FDTD) simulation was used to numerically calculate the light field distribution around scratches on rear surface (i.e. exit surface for light) which indicates that modulated light intensity is susceptible to the profile and size of scratches. FDTD simulation results also indicate that the light field intensification is elevated with the dimension of scratches and light modulation effects in triangular scratches are usually not as notable as serrated and parabolic scratches.

## Introduction

Fused silica is widely used in large-aperture high power laser systems as it can transmit a wide spectrum of light from ultraviolet to infrared wavelength. When exposed to 351/355 nm (UV) laser, fused silica may be damaged on the surface at much lower fluence <10 J/cm^2^ than intrinsic breakdown threshold ~100 J/cm^2^, which has been a main barrier to constructing high power/energy giant laser systems^1–5^. The causes for such a low laser-induced damage threshold (LIDT) are ascribed to mechanical and/or chemical defects incurred during the manufacturing of optical components, e.g. scratches/cracks and contaminations^4–9^. P. Cormont found that the damage resistance of fused silica decreases with the width of polishing-induced scratches, the LIDT on unscratched area was 24 J/cm^2^@3 ns while the LIDT at scratches wider than 30 µm asymptotically dropped to 5 J/cm^2^@3 ns^7^; our previous research revealed that ductile scratches also play a perceptible role in laser induced damage and the damage density is greatly increased about one order of magnitude because of the ductile and brittle scratches^8^. The possible reasons for the damage aggravation resulted from scratches could be the light field modulation triggered by the scratches, the presence of absorbing substance dormant in the scratch, or the weakness of the material because of existing mechanical flaws^4–6^. Some research findings show that light field enhancement is a major cause for laser damage ignition^10,11^. In this paper, scratches were intentionally brought about during polishing process by infiltrating foreign abrasives into various ceria-based slurries: the mixture of ceria and rough SiC W7/W40 at diverse weight concentrations. The polishing-induced scratches fall into two categories: ductile and brittle ones. Brittle scratches will be produced by spiking large abrasives W40 and small abrasives W7 may only generate ductile scratches. Furthermore, the augment in size and/or the concentration of rough particles leads to more scratches. Ductile and brittle scratches are different in profile and dimension but they both deteriorate the damage resistance of fused silica optics. The possible reasons for damage initiation to fused silica are explored in this paper and the finite difference time domain (FDTD) algorithm is used to simulate how the polishing-induced scratches can modulate the light field of incident laser.

## Experimental Preparation

### Samples manufacturing

**Figure 1:**
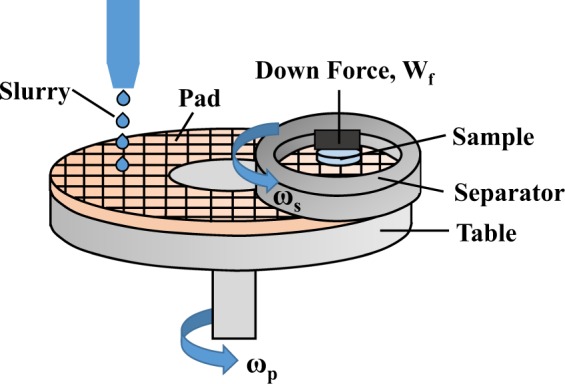
The schematic diagram of the lapping set-up. The polyurethane pad was adhered onto a synthetic tin plate and the sample was located in a separator. The platen and the separator were driven independently.

Fused silica samples (50 mm in diameter and 5 mm thick) were polished using the lapping machine (FD-380XL, Fonda, China). The polyurethane pad was adhered onto a synthetic tin plate and the samples were located in a separator. The polyurethane pad used is manufactured by Universal Photonics Inc., USA. A dead weight load of ~2.9 N is applied onto the samples. During the polishing process, both the separator and the tin plate were driven independently and their rotation speed ratio is 50:1, and the slurries were fed continuously at a flow rate of 10 mL/min. The schematic diagram of the lapping set-up is shown in Fig. 1. Four polishing slurries were used to polish fused silica, including A: ceria ~0.3–0.5 μm in diameter, B: the mixture of ceria and rough abrasives SiC W7 (diameter ~7 μm), 2:1 in mass concentration, C and D are composed of ceria with SiC W40 additives (diameter ~40 μm), and their mass concentration is 20:1 and 2:1, respectively.

### Surface characterization

The surface morphology of polished samples was observed using an in-house built defect detecting system and the testing spot size is ~14.8 × 14.8 mm^2^. The section profile of scratches was obtained using an atomic force microscope (AFM; Bruker UPTI-150, Germany) and the sampling size of each testing site is 30 × 30 μm^2^. An optical microscopy (Keyence VHX-2000, Japan) was used to inspect the morphologies of damaged area prior to and following laser illumination.

### Laser damage testing

**Figure 2:**
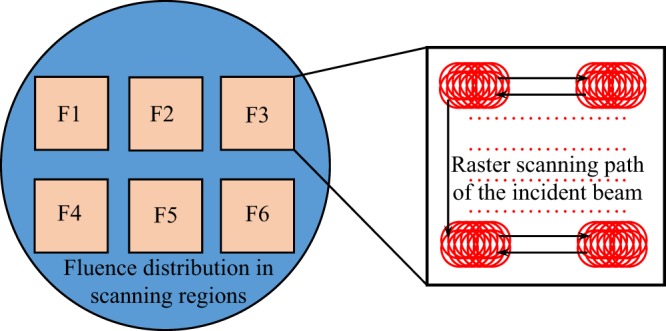
The schematic diagram of raster scanning damage testing protocol. The adjacent laser spots in each region overlapped 90% in radial direction.

The tripled frequency 3ω Nd:YAG laser damage testing system (Laser Zentrum Hannovere.V., Germany) was used to evaluate the damage performance of polished samples adopting raster scanning testing protocol. The exist surface of sample was perpendicularly subjected to a Gaussian laser pulse (8 ns@355 nm, beam waist 800 μm) at the repetition rate of 10 Hz, and the irradiated area was inspected by a long-focus microscope equipped with a CCD camera (resolution ∼10 μm) to record damage initiation. The magnification of the microscope is adjustable from 0.7–4.5 and the view field of the objective is ~0.5–3 mm. In raster scanning testing, the surface of each sample was divided into 3–6 sub-regions (dimension 10 mm × 10 mm), and each sub-region was sufficiently irradiated with a fixed laser fluence. The stage of sample holder moved at a predetermined speed ~8 mm/s to ensure each laser shot overlapped ~90% in area with adjacent shot, as illustrated in Fig. 2. The detailed layout of damage testing system is shown in references^12–14^. Our ultimate goal is to investigate the damage performance of large-aperture optics for Nd:glass laser (tripled frequency 351 nm, 3 ns), so the damage threshold laser fluence is rescaled from 8 ns to 3 ns in the article.

## Experimental Results

### Surface morphology of polished sample

**Figure 3:**
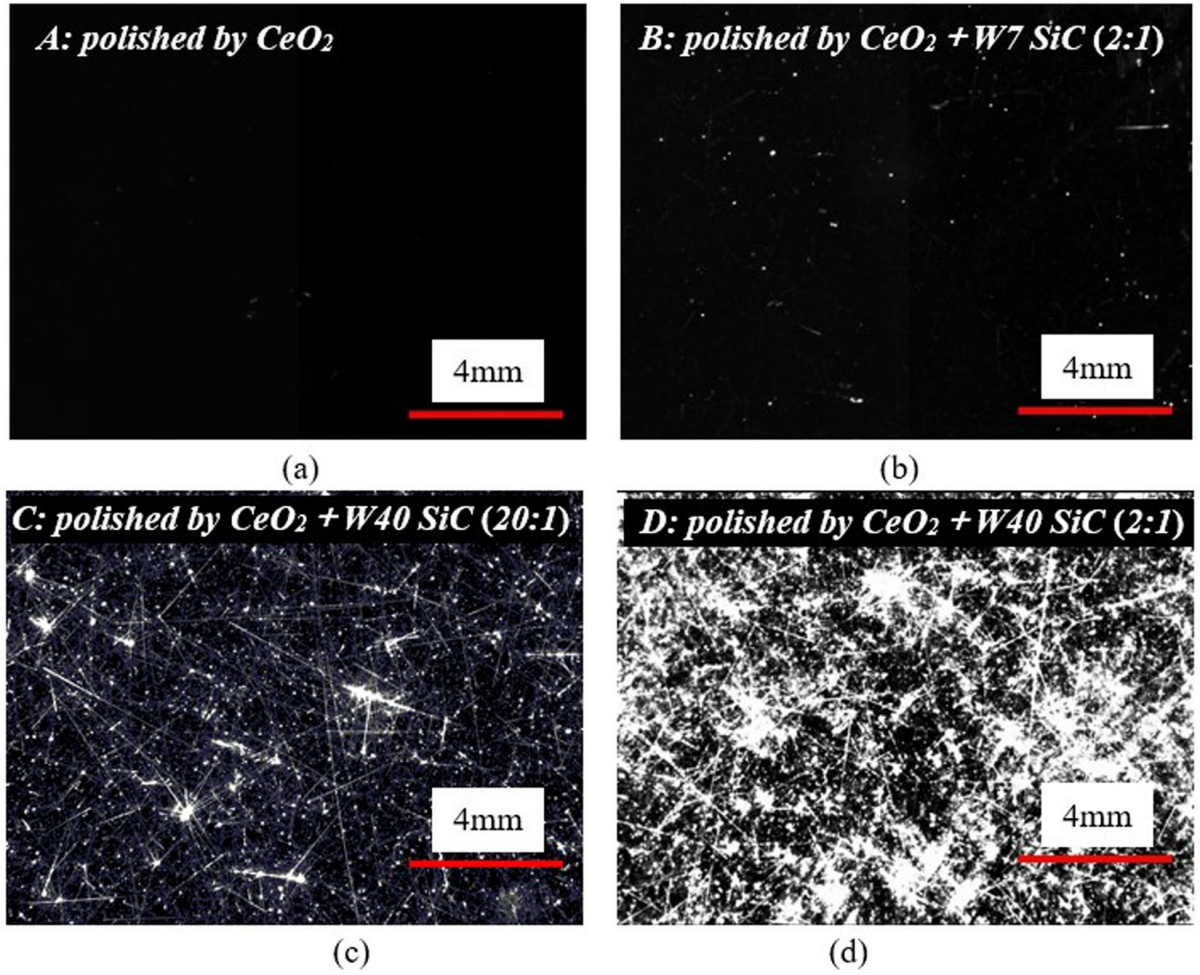
Surface morphologies of four polished samples. No obvious scratches are found on CeO_2_-polished sample A, and the observed scratches on sample B, C and D are caused by the rough particles SiC W7 and/or W40 addition. Weight ratio of CeO_2_ and SiC is 2:1 or 20:1.

The surface morphology of polished samples is shown in Fig. 3, and it is observed using a surface defect detecting system. The slurry of sub-micron CeO_2_ can yield fused silica of scratch-free surface (sample A). In contrast, various types of scratches is found on sample B, C and D as a result of the addition of rough particles SiC W7 or W40, as Fig. 3(b–d) show. These scratches can be divided into two basic categories^15^: (1) Ductile scratches that show no brittle fractures but just plastic modification to the surface; (2) Brittle scratches that have accompanied cracks. From Fig. 3(a), the surface of sample A that shows no scratches or digs can be created using our polisher with ~0.3–0.5 μm CeO_2_ slurries. After adding SiC a kind of harder and larger abrasive than CeO_2_, many scratches get visible (Fig. 3(b–d)). The surface contains several ductile scratches following polishing with the adulteration of CeO_2_ and ~7 μm SiC (mass concentration 2:1), and there were few brittle scratches on the surface (sample B, Fig. 3(b)). The material of sample C and D is seriously fractured and the abovementioned two types of scratches are both generated when fused silica are processed using CeO_2_ doped with ~40 μm SiC (Fig. 3(c,d)). Moreover, the scratches including brittle and ductile are densified significantly when increasing the concentration of SiC. The reason for the appearance of brittle scratches when larger SiC abrasives W40 were mingled into CeO_2_ slurry is that increasing the size of SiC abrasives will reduce the number of abrasives bearing the downward load thus the load on a single abrasive will increase accordingly, and brittle fractures would be expected where the load exceeds a critical load of material to induce brittle scratches. The area scale of surface scratches was statistically evaluated using an image analysis software, in which the images of scratched samples were first binarized into white-black images and then the ratio of the scratch pixels to the whole pixels was evaluated to quantify the scale of scratches, thus the surface morphology in micron to sub-micron dimension can be captured. The results are shown in Table 1 and we can see clearly that increasing the size and/or the concentration of additive particles would lead to severer scratching. The addition of W7 ~7 μm into ceria-based slurries can generate only ductile scratches that account for ~2.31% area of the polished surface area on sample B. When larger particles W40 ~40 μm were employed during polishing, both ductile and brittle scratches will be produced. Upon increasing the concentration of SiC, and their area proportion are remarkably increased to ~30.08% and ~2.25% on sample C and ~47.85% and ~10.93% on sample D, respectively, as compared to sample A and B.

**Table 1:** Scratches distribution on samples polished with various slurries.

Sample	Slurry	Ductile scratches				Brittle scratches			
		Scale	Width (μm)	Depth (nm)	Profile*	Scale	Width (μm)	Depth (nm)	Profile*
A	CeO_2_	~0	/	/	/	0	/	/	/
B	CeO_2_ + SiC W7 (2:1)	2.31%	0.2–1	5–10	T	~0	/	/	/
C	CeO_2_ + SiC W40 (20:1)	30.08%	1–2	10–60	T	2.25%	2–4	80–200	S, P
D	CeO_2_ + SiC W40 (2:1)	47.85%	~1	10–20	T	10.93%	1–4	20–120	S, P

Increasing the particle size or the concentration of SiC will give rise to more scratches. The shape and size of scratches differ between the ductile and brittle scratches, and they are at most ~4 μm wide and ~200 nm deep.

*The profile of T, S and P represents the shape of triangular, serrated and parabolic, successively.

**Figure 4:**
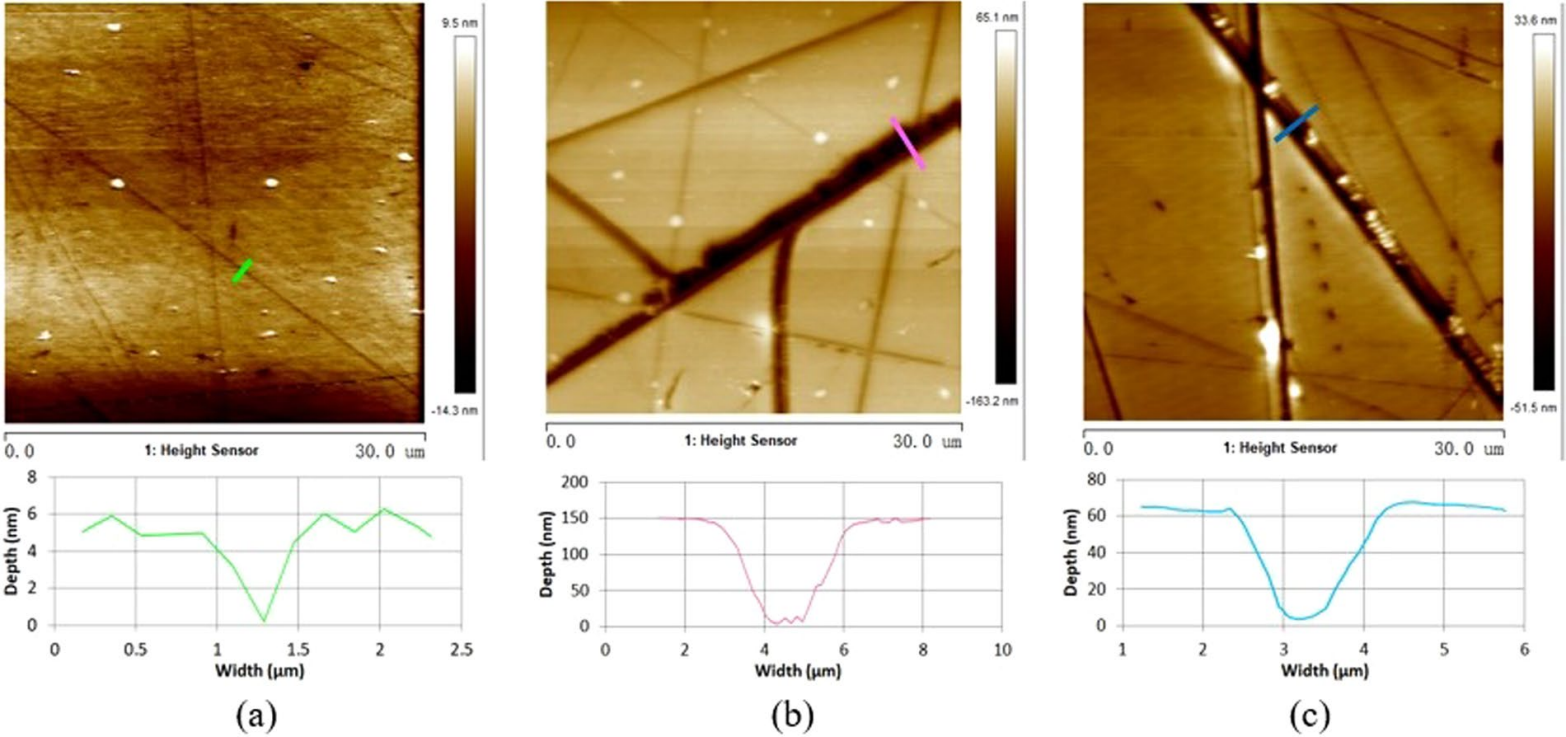
Atomic force microscopic images of scratches on samples (**a**) B (**b**) C and (**c**) D. The ductile scratches in (**a**) contain plastic deformation and have a shape of triangle; the profile of brittle scratches in (**b**,**c**) shows serrated and parabolic, with accompanied fractures.

The atomic force microscopic images in Fig. 4 reveal that both the profiles and dimensions of scratches vary as the type of scratch changes. The white spots in the images may be resulted from noises during measurement. The ductile scratches on sample B following CeO_2_ + W7 (2:1) polishing show smooth edge and bottom along the path with a triangle profile of ~0.5 μm in width and ~5 nm in depth (Fig. 4(a)). It follows from Fig. 4(b,c) that brittle scratches have more anomalies in morphology. The section of brittle scratches are with serrated bottom (~3 μm wide and ~150 nm deep, Fig. 4(b)) and/or approximately parabolic (~2 μm wide and ~60 nm deep, Fig. 4(c)). The larger abrasive particles used during polishing processing will bear a much higher load than the average particle on the lap when they move on the fused silica, and plastic type scratches would be generated when the local load of abrasive exceeds the yield stress at the contact zone, and brittle type scratches would be originated at a higher load^15^. During the polishing processing of fused silica optics, the compressive pressure is formed due to the contact between the material surface with spherical abrasives, such as CeO_2_^8^, and the pressure is approximately spherically symmetric. If the abrasives continue to scratch along the direction perpendicular to the radius of sphere, the transverse scratches with a triangular profile will be generated. Regarding the polishing abrasives have sharp edges, such as W7 or W40 SiC^8^, the contact surface between the abrasives and material tends to be polyhedral, and the iso-stress surface will be distorted when such abrasives slide at a single direction and finally resulting in the brittle fractures with serrated morphologies. Moreover, parabolic scratches would be expected when the sharp abrasives such as W7 or W40 SiC bear unevenly distributed loads since their edges are in contact with fused silica surface, which is similar to the “jumping” polishing on the surface. Furthermore, scratches on polished surfaces were profiled and their morphology characteristics are summarized in Table 1. On sample B, C and D, the ductile scratches mostly feature triangle in section shape and their transverse size vary within 0.2–2 μm and 5–60 nm, respectively. Both serrated and parabolic profiles of brittle scratches can be found on sample C and D, which are usually larger in dimension than ductile scratches, ranging from 1 to 4 μm in width and no more than 200 nm in depth.

### Laser damage performance

**Figure 5:**
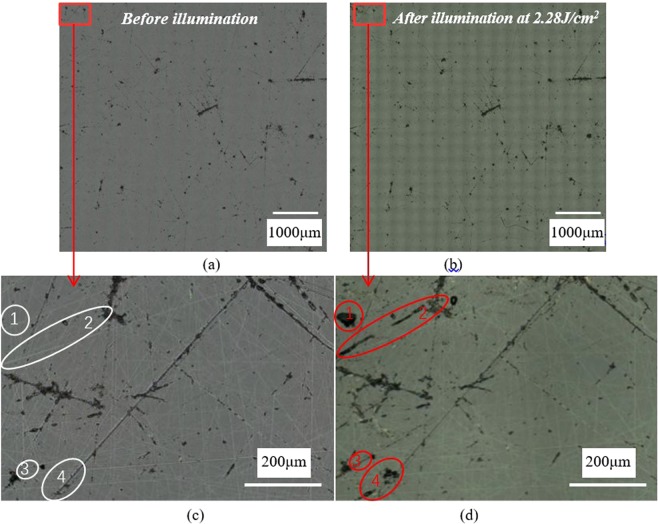
Surface of sample D (**a**,**c**) before and (**b**,**d**) following laser raster scanning with 2.28 J/cm^2^, both brittle and ductile scratches can lead to damage (as shown in red circles).

The polished samples were scanned by laser pulses of various energy fluences (8 ns, 355 nm) and the fluence was then rescaled to 3 ns using empirical rule^14^. Figure 5 demonstrates the surface morphology of sample D (polished with CeO_2_ + W40 (2:1)) before and after exposure to 2.28 J/cm^2^ laser. From Fig. 5(d), field 4 was seriously damaged after 2.28 J/cm^2^ laser illumination and there are brittle scratches in this field before damage testing (Fig. 5(c)). In spite of only ductile scratches in field 1&2&3, damage also happened after raster-scan testing in the fields. It can be known clearly that both plastic deformation and fractured cracks deteriorate damage resistance of optics.

**Figure 6:**
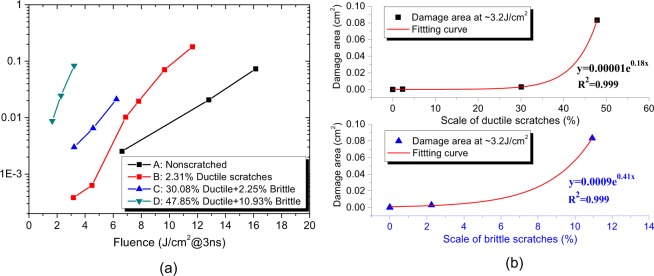
(**a**) Damaged area tends to increase with the laser fluence, and increasing scratching is more detrimental to damage resistance of fused silica. (**b**) Damage area at ~3.2 J/cm^2^ increases exponentially with the proportion of both ductile and brittle scratches. Brittle scratches are easier to be damaged than ductile scratches due to the greater fitting coefficients.

Following the laser raster scanning, online microscopic image system (resolution ∼10 μm) was enabled to identify new and grown defects by comparing the pre-image and current image at the same place during different scanning regions. And the damage density can be extracted from the defects density before and after testing^8,16,17^. In view of a great number of scratches existing on sample C (30.08% ductile mixed with 2.25% brittle) and D (47.85% ductile mixed with 10.93% brittle), the microscopic images before and after laser scanning covered with a myriad of defects the damaged area was determined using the pixel discrepancy of defects before and after laser irradiation to quantitatively evaluate the damage performance of polished samples. The damaged area versus incident fluence was plotted in Fig. 6(a), it can be known that samples are prone to damage under high laser fluence and damaged area was increased by one to two orders of magnitude on scratched samples in comparison to unscratched surface. Upon ~7 J/cm^2^ laser illumination, ~2.5 × 10^−3^cm^2^ area was damaged on defect-free sample A, and the damaged area is measured to be 0.01 cm^2^ on sample B with ~2.31% ductile scratches, revealing that ductile scratches degrade the damage resistance of fused silica. Following ~3.2 J/cm^2^ laser irradiation, damage sites in total area of ~3.8 × 10^−4^cm^2^ were detected on sample B, and much larger damaged area was ignited on sample C ~0.003 cm^2^ and sample D ~0.08 cm^2^, indicating that increased proportion of scratches on the surface is responsible for the worse damage resistance. It can be concluded that even the ductile scratches with no fractures but just plastic modification can limit the damage performance of fused silica, and the results seem different from that in ref.^18^, in which little evidence was found that either displaced or densified material plays a significant role in optical damage threshold. The inconformity between our work and the previous reports may be related to the different methods we applied to generate the local flaws and the different types of flaws we investigated. We generated scratches by polishing instead of using an indenter, and the continuous scratches were investigated in this paper instead of indentation. Figure 6(b) plots the relationship between the scale of ductile/brittle scratches with the damaged area following ~3.2 J/cm^2^ laser scanning. Damaged area at ~3.2 J/cm^2^^ 2^ is found to increase exponentially with the scale of surface scratches. Damage performance of fused silica heavily relies on the appearance of brittle scratches, because the fitting coefficients for brittle scratches ~0.0009 and ~0.41 are greater than the counterparts of ductile scratches ~0.00001, ~0.18, respectively.

## Discussion

Surface scratches are responsible for electric/light intensification near fused silica when exposed to UV laser pulses, which is a possible reason for the initiation or catalysis of laser induced damage^10,11^. It has been reported that the optics are more likely to damage when the scratches were located on the rear surface than front surface^6,19^, and thus the localized electric/light enhancement around scratches on the rear surface is discussed in detail in this paper. The electric/light field distribution around scratches was simulated using analysis software on the basis of finite difference time domain (FDTD) and the two-dimensional model of FDTD was set up. FDTD method is based on iterative solution to Maxwell’s equations and the distribution of electric field and magnetic field within the space can be solved gradually and alternately^19,20^.

**Figure 7:**
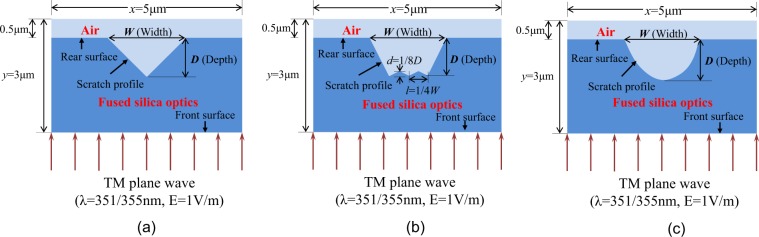
Three types of scratch models in FDTD simulation. (**a**) Triangular scratch, (**b**) Serrated scratch and (**c**) Parabolic scratch. All the scratches are situated on the rear surface of optics and the incident light is TM plane wave (wavelength ~355 nm, electric field ~1 V/m).

The two-dimensional models for simulation are demonstrated in Fig. 7, in which three corresponding types of scratches models: triangular, serrated and parabolic were established according to the profiles in Fig. 4. A TM plane wave is normally incident on the front surface and its wavelength is 355 nm and the electric field value is 1 V/m. For fused silica optics, its relative dielectric constant and refractive index are 2.25 and 1.48, and scratches are thought to be filled with air whose refractive index is 1. The lateral size *W* and depth *D* of scratches can be adjusted with calculation requirements; as to serrated scratches, the jagged structure at the bottom has a periodic spacing *l* and height *d*, which are fixed to be a quarter of *W* and one-eighth of *D*, respectively (Fig. 7(b)). The simulation domain is set as *x*(5 μm) × *y*(3 μm) area, and the rear surface of silica is covered with a 0.5 μm air layer. A benefitting absorption boundary condition needs to be set in order to simulate the wave propagation within the infinite space. In our calculation, the perfectly-matched-layer (PML) boundary is applied for the left and right borders and scattering boundary for the up and down borders, because these boundaries can absorb the light wave effectively and never cause obvious reflection of the wave^21,22^.

**Figure 8:**
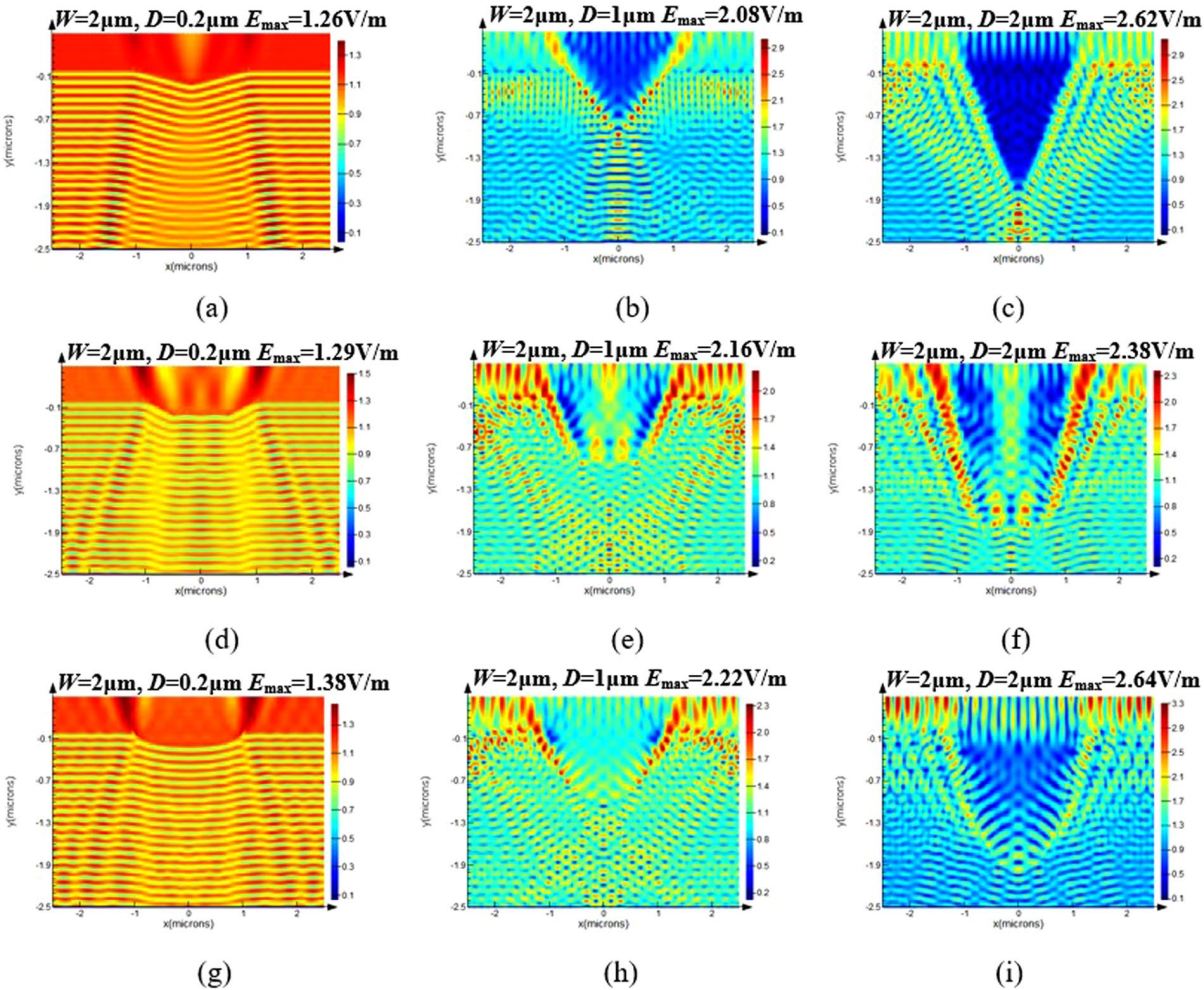
Electric field distribution around diverse rear scratches (**a**–**c**) triangular, (**d**–**f**) serrated and (**g**–**i**) parabolic scratches. Electric field amplitude *E*_*max*_ increases with the depth of scratches, and parabolic scratches enhance the electric field most significantly.

When the incident light (wavelength ~355 nm, electric field ~1 V/m) goes through both surfaces of glass, the electric field modulated by the rear scratches can be resolved. Figure 8 demonstrates the electric field distribution around diverse profiles of scratches (a–c) triangular scratches (d–f) serrated scratches and (g–i) parabolic scratches, among them the triangular profiles represent the ductile scratches, and serrated and parabolic ones are representatives of brittle scratches. Due to the existing of rear surface scratches, the constructive interference between the incident beam and reflection beam may lead to the modulation of electric/light field near the scratches. Thus the shape, size and the position of scratches, the incident angle and the wavelength of beam, and the polarization of electric field, etc. are influential to the light distribution inside the optics elements. Regarding triangular scratches, the incident beam will reflect and transmit at the two hypotenuse of the scratch, and the interference of incident light and reflected light in the lower half space may lead to the local light field intensification on both sides near scratch-air interface and the region directly below the scratch. Therefore, the inclination angle of triangular scratches, namely the angle between the scratch and the normal direction of the exit plane will affect the reflective and transmission condition of incident beam and ultimately affect the light distribution inside the element. The serrated scratch has a relatively constant inclination angle on the two inclined planes on both sides, and the uneven structure at the bottom can result in multiple reflection of incident laser at the scratch-air interface and the interference and superposition of incident light and reflected light, so as to enhance the light distribution inside fused silica optics. In terms of parabolic scratches, the irradiation beam will transmit at the bottom because of the small incidence angle at the bottom, and irradiation beam is prone to be reflected at the relatively distant location on both sides of scratches since the incidence angle is greater. Moreover, the reflect light near surface tends to give rise to secondary total reflection in the horizontal interface, and the superposition of primary and secondary total reflection beams with the incident beam will result in light field intensification near the surface of parabolic scratch^22^. The investigated scratches in Fig. 8 have a constant width *W* = 2 μm, and their depths *D* are 0.2, 1 and 2 μm, respectively. For the three profiles of scratches, the electric field amplitude near the back surface *E*_*max*_ is found to enhance significantly with the increase of scratch depth. Taking triangular scratch an example, *E*_*max*_ is raised from 1.26 V/m to nearly double 2.62 V/m when the depth increases from 0.2 μm to 2 μm. Moreover, the electric field modulation is sensitive to the shape and type of scratches. For similar size of 2 μm wide and 0.2um deep, *E*_*max*_ near the serrated and parabolic scratches 1.29 V/m and 1.38 V/m are higher than that of triangular scratches 1.26 V/m.

The simulation analysis of electric field modulation was also carried out on the ideal and non-scratched glass surface, and the peak value of electric field *E*_*max*_ near the rear surface is simulated to be ~1.19 V/m. The simulation result coincides with the theoretical calculation, which shows that the ratio of the electric field value of transmission wave *E*_*t*_ to that of incident wave *E*_*i*_ takes the form of the following equation^19^:

1 $$\frac{{E}_{t}}{{E}_{i}}=\frac{2{n}_{1}}{{n}_{1}+{n}_{2}}$$

where *n*_1_ = 1.48 and *n*_2_ = 1 represent the refractive index of glass and air, respectively. When the electric intensity of input light *E*_*i*_ is 1 V/m, the theoretical results of the electric intensity for transmission light on output surface *E*_*t*_ is ~1.19 V/m.

**Figure 9:**
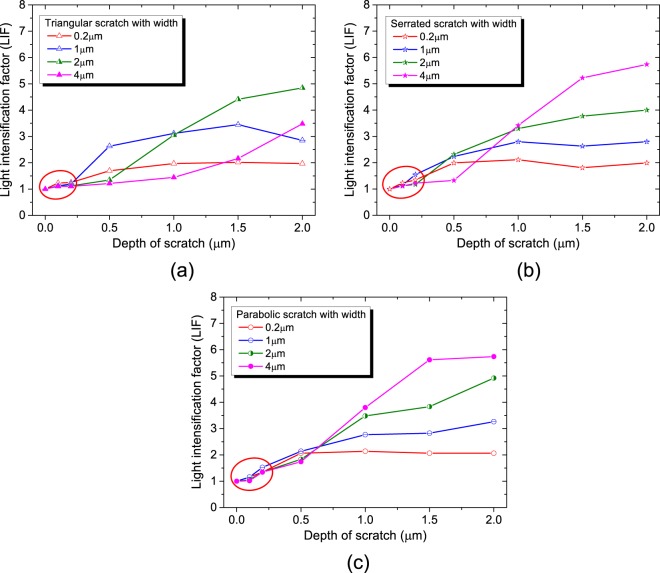
Light intensification factor (LIF) are usually greater in (**b**) serrated and (**c**) parabolic (brittle) scratches than (**a**) triangular (ductile) scratches. LIF increases with the size of scratches and it is less than 1.5 for the polishing-induced scratches within the depth 200 nm (red circle indicates).

The light intensity near rear scratches was investigated as prescribed by Fresnel’s law that the light field intensity *I*_*i*_ and the electric field intensity *E*_*i*_ meets the equation ${I}_{i}={{E}_{i}}^{2}$ in a single medium^20,23^. In order to evaluate the light modulation resulted from rear scratches and explore their damage performance influence, the light intensification factor (LIF) is defined as:

2 $${LIF}=\frac{{I}_{{\max }}}{{I}_{0}}$$

where *I*_0_ is the output light intensity of a defect-free bulk ~1.41(*I*_0_ = 1.19^2^), and *I*_max_ is the peak value of light intensity modulated by scratches, in which high LIF usually means more proneness to laser damage resistance^10^. The light intensification factor around various polishing-induced scratches is summarized in Fig. 9. According to previous data, the triangular, serrated and parabolic scratches with the widths 0.2 μm, 1 μm, 2 μm and 4 μm are under discussion and their depth ranges from 0 to 2 μm. We can see clearly from Fig. 9 that LIF of large scratches (width 2 μm/4 μm) has multiplied as the depth increases. The LIF of triangular, serrated and parabolic scratches (width 4 μm) was intensified from ~1.10 to ~3.48, ~1.02 to ~5.74 and ~1.11 to ~5.74, respectively, when their depth increases from 0.1 to 2 μm (Fig. 9(a–c)). Meanwhile, the broadening in lateral size of scratches can also lead to LIF elevation. At the depth of 2 μm, the LIF of triangular scratch is 1.97 and 3.48 when its width is 0.2 μm and 4 μm, respectively (Fig. 9(a)). Moreover, the LIF of triangular scratch is basically not so high as the other two shapes of scratches at similar sizes, indicating that light field is intensified more dramatically in brittle scratches that are usually serrated bottom and/or parabolic in profile than ductile scratches that are usually triangular in profile. This result may account for more marked deterioration of damage performance resulting from brittle scratches.

Table 1 has suggested that the polishing-induced scratches in this paper were not deeper than 200 nm. From Fig. 9 it can be known that the LIF of scratches within 0.2 μm in depth is 1.5 at most, as the red circles indicate. The marginal light intensification is too limited to damage fused silica, indicating that the primary cause for laser damage in this kind of surface may be some factors other than light field modulation, such as coupled absorption of light owing to absorbers (chemical inclusions, electronic defects, etc.) and light intensification^24–26^, which needs further discussion.

## Conclusion

The scratches were investigated in order to find out their possible effects on the laser damage performance of fused silica and the light field distribution under laser illumination was modelled. The results show that brittle scratches can be induced when large sized abrasives (W40, diameter ~40 μm) were added into ceria-based polishing slurries and smaller rough abrasives (W7, diameter ~7 μm) may only generate ductile scratches. Increasing the concentration and/or the size of rough particles will definitely raise the proportion of surface scratches. The profile and the size of scratches are found to vary with the types of scratches and ductile scratches are usually smooth with triangular profile and their width vary in the range ~0.2–2 μm and depth ~5–60 nm whilst brittle scratches which show irregularity along the path are usually with serrated bottom and/or parabolic in profile and have greater dimensions ~1–4 μm in width and ~20–200 nm in depth. The raster scan damage testing results reveal that both ductile and brittle scratches have great impacts on damage resistance since the damage area was increased about one to two orders of magnitude relative to unscratched surface and brittle scratches are more deleterious to the optics than ductile scratches. FDTD simulation results indicate that the light field intensification is varied with the depth and the width of scratches and light modulation effects in triangular scratches are usually not as notable as serrated and parabolic scratches. The light enhancement factor is not more than 1.5 when the scratches are below 200 nm in depth. Other factors like coupled absorption of light owing to absorbers (chemical inclusions, electronic defects, etc.) and light intensification may be responsible for the laser induced damage at the scratches on fused silica.
